# Using machine learning to predict five-year transplant-free survival among infants with hypoplastic left heart syndrome

**DOI:** 10.1038/s41598-024-55285-1

**Published:** 2024-02-24

**Authors:** Andrew H. Smith, Geoffrey M. Gray, Awais Ashfaq, Alfred Asante-Korang, Mohamed A. Rehman, Luis M. Ahumada

**Affiliations:** 1 Division of Cardiac Critical Care Medicine, The Heart Institute, Johns Hopkins All Children’s Hospital, 501 6th Avenue South, St. Petersburg, FL 33701 USA; 2 Center for Pediatric Data Science and Analytic Methodology, Johns Hopkins All Children’s Hospital, St. Petersburg, FL USA; 3 Cardiovascular Surgery, Heart Institute, Johns Hopkins All Children’s Hospital, St. Petersburg, FL USA; 4 Heart Transplantation, Cardiomyopathy and Heart Failure, Heart Institute, Johns Hopkins All Children’s Hospital, St. Petersburg, FL USA; 5 Department of Anesthesia and Pain Medicine, Johns Hopkins All Children’s Hospital, St. Petersburg, FL USA

**Keywords:** Cardiovascular Surgical Procedures, Heart Defects, Congenital, Intensive Care Units, Pediatric, Infant, Newborn, Disease, Paediatrics, Cardiology

## Abstract

Hypoplastic left heart syndrome (HLHS) is a congenital malformation commonly treated with palliative surgery and is associated with significant morbidity and mortality. Risk stratification models have often relied upon traditional survival analyses or outcomes data failing to extend beyond infancy. Individualized prediction of transplant-free survival (TFS) employing machine learning (ML) based analyses of outcomes beyond infancy may provide further valuable insight for families and healthcare providers along the course of a staged palliation. Data from both the Pediatric Heart Network (PHN) Single Ventricle Reconstruction (SVR) trial and Extension study (SVR II), which extended cohort follow up for five years was used to develop ML-driven models predicting TFS. Models incrementally incorporated features corresponding to successive phases of care, from pre-Stage 1 palliation (S1P) through the stage 2 palliation (S2P) hospitalization. Models trained with features from Pre-S1P, S1P operation, and S1P hospitalization all demonstrated time-dependent area under the curves (td-AUC) beyond 0.70 through 5 years following S1P, with a model incorporating features through S1P hospitalization demonstrating particularly robust performance (td-AUC 0.838 (95% CI 0.836–0.840)). Machine learning may offer a clinically useful alternative means of providing individualized survival probability predictions, years following the staged surgical palliation of hypoplastic left heart syndrome.

## Introduction

Hypoplastic left heart syndrome (HLHS) results from the underdevelopment of a series of left-sided cardiac structures including mitral and aortic valves, aortic arch, and left ventricle. Uniformly fatal if not addressed in the neonatal period, current approaches to therapy for HLHS most frequently rely upon palliative surgical or catheter-based interventions. The most frequently-employed of these early palliative approaches is the stage 1 (Norwood) procedure (S1P), which ultimately relies upon a single ventricle providing cardiac output for both pulmonary and systemic circulations through a reconstructed aorta along with a shunt between the systemic circulation and pulmonary arteries^1^. Following the S1P, infants subsequently experience an “interstage” period followed by a stage 2 palliation (S2P) consisting of shunt removal and creation of an anastomosis between superior vena cava and pulmonary arteries, typically between three and six months of age. While HLHS accounted for nearly a quarter of all neonatal deaths in the United States due to congenital heart disease annually in the twentieth century, mortality in infancy remains in excess of 10% despite advances in management over the past three decades, particularly among infants with comorbid conditions^2,3^.

The Pediatric Heart Network (PHN) Single Ventricle Reconstruction (SVR) trial was a randomized, controlled trial assessing two approaches to the provision of pulmonary blood flow associated with the S1P, with the resultant deidentified data made publicly available at the conclusion of the trial^4^. A follow-up study to the SVR trial longitudinally followed study participants up to 6 years of age, through second and third-staged palliations, known as the Single Ventricle Reconstruction Extension Study (SVR II). Results from this study demonstrated significant morbidity and mortality over the study follow up period, with transplant free survival (TFS) of approximately 60% in the study cohort^5^. While investigators to date have applied machine learning (ML) algorithms to SVR trial data in order to more accurately predict one-year TFS among its study participants relative to previous analyses, there remains the need for an instrument that may describe an individualized prediction of death or transplant beyond infancy into childhood, accounting for the cumulative and nonlinear effects of risk factors over successive phases of care^6^. The purpose of this study was to leverage the longer endpoint data of the SVR II study to inform an ML-based model, which can accurately predict individualized transplant-free survival through five years using data available prior to S1P. A secondary outcome of interest consisted of developing an adaptive model which adjusts inputs based upon a patient’s course of care, through S2P hospitalization. An individualized, ML-driven instrument applied to successive phases of care of a complex staged palliation may in turn serve as a valuable decision-making tool for providers and families alike.

## Results

**Figure 1:**
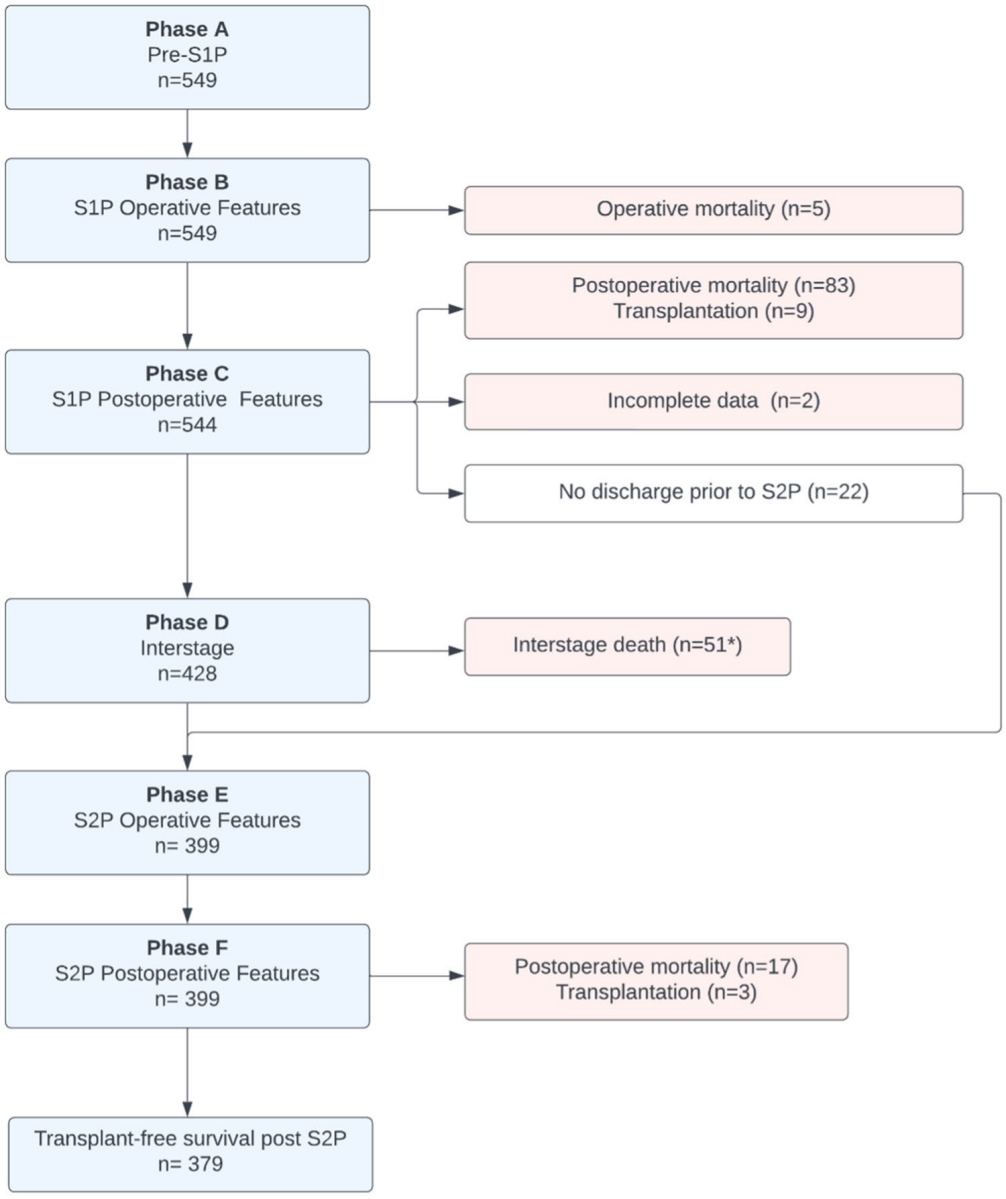
Sequential phases of care associated with the palliative management of hypoplastic left heart syndrome during the Single Ventricle Reconstruction trial, with associated numbers of study participants employed in model development.

As summarized in Fig. 1, features for consideration within the model were selected along sequential time points along the course of care, from Phase A (pre-S1P) through hospitalization following S2P (Phase F). Of the 549 patients undergoing S1P, 212 (39%) patients met the primary endpoint of either death or transplant, with a median follow up time in this series of 5.9 (interquartile range 0.4–6.1) years. Progression of study participants included for consideration beyond Phase A is summarized in Fig. 1, with TFS noted in 69% (n = 379) by the conclusion of Phase F. Notably there were 22 patients not discharged prior to S2P and were excluded from the Phase D model. Two subjects were also excluded from modeling beyond Phase C due to data incompleteness. Additionally, of 51 deaths during the interstage phase (Phase D), one occurred beyond the endpoint of the original SVR trial.

### Model performance

Performance metrics for the top models are summarized in Table 1.

**Table 1:** Top-performing models and metrics with respect to phase of care.

Phase	Features	C-Index [95% CI]	Brier Score [95% CI]
A	45	0.692 [0.689,0.695]	0.195 [0.194, 0.197]
B	50	0.695 [0.692, 0.698]	0.194 [0.193, 0.195]
C	100	0.822 [0.820, 0.825]	0.124 [0.123, 0.125]
D	120	0.626 [0.621, 0.630]	0.103 [0.102,0.105]
E	150	0.665 [0.659, 0.671]	0.101 [0.099, 0.102]
F	195	0.620 [0.614, 0.625]	0.096 [0.085, 0.088]

The Concordance index and Brier scores of the test models are shown with 95% confidence intervals.

**Figure 2:**
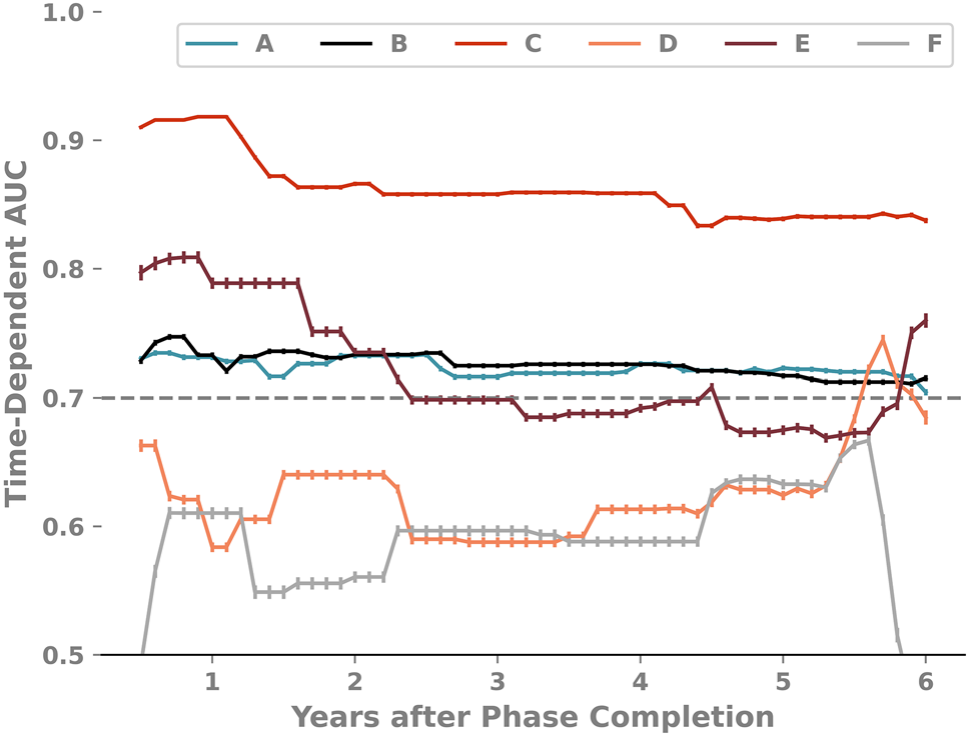
Summary of time-dependent area under the curve (td-AUC) performance of models predicting transplant-free survival trained with features available during sequential phases of care, from the pre-S1P phase (Phase A) through hospitalization following S2P (Phase F). A model incorporating features through S1P hospitalization (Phase C) demonstrated superior performance over the five year study period (C index 0.822 [95%CI 0.820–0.825]). *S1P* Stage 1 palliation, *S2P* Stage 2 palliation, *td-AUC* time-dependent area under the curve.

The Brier score for the test set approached 0 with subsequent clinical phases, with Phase A demonstrating a value of 0.195 and Phase F yielding a value of 0.096. The td-AUC (Fig. 2) shows good performance for Phases A through C, all demonstrating stable performance and a td-AUC > 0.70 across the entire study period; Phase C was the highest performing model, with a td-AUC > 0.80 across the entirety of the five year study period. While Phase E demonstrated a notable decrement in performance in the first two years following S2P, phases D and F were the most variably performing models with td-AUC largely below 0.70 for the entirety of the study period.

### SHAP values

**Figure 3:**
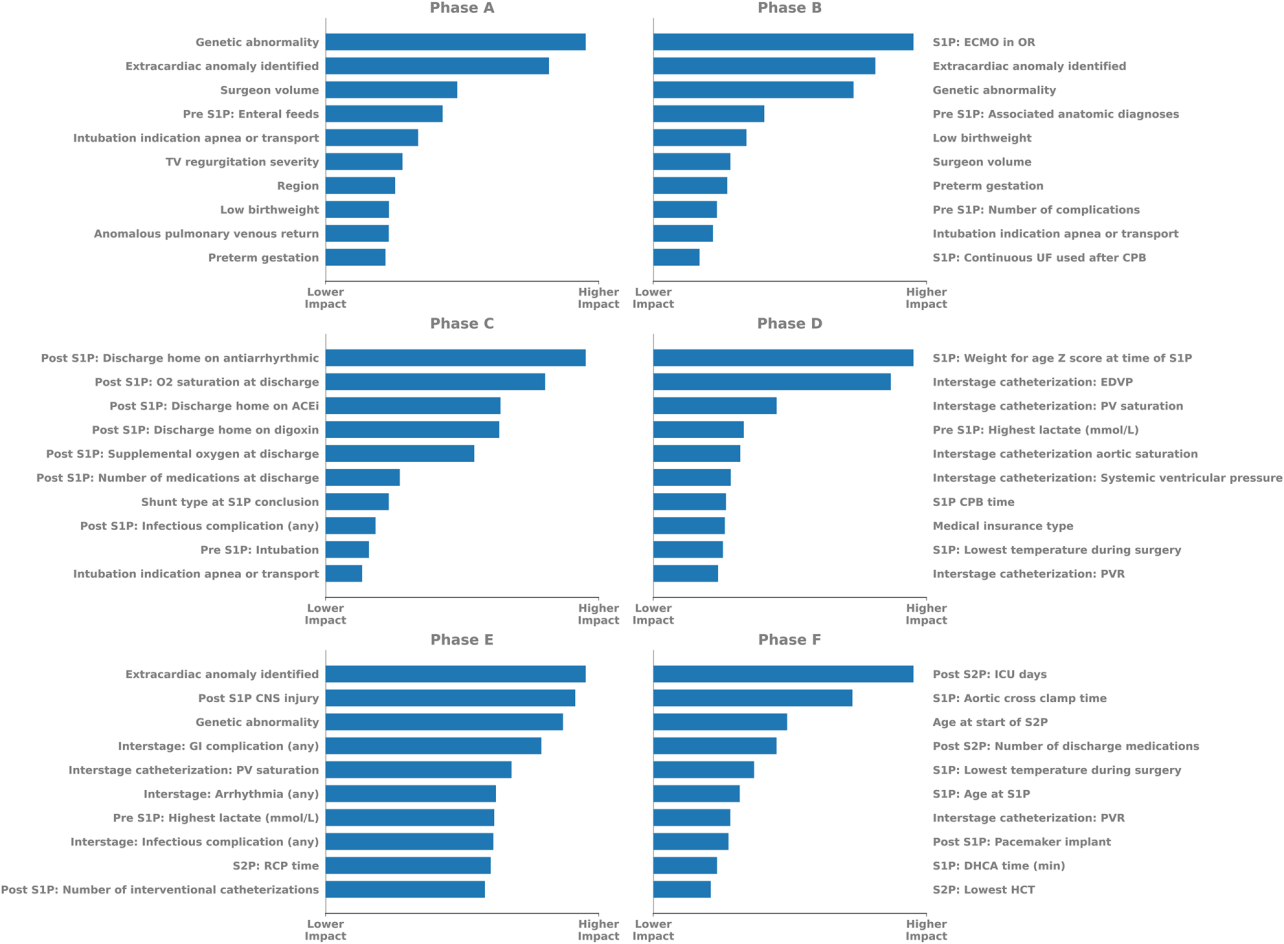
Bar plots showing mean SHAP values for the 10 most important features for Phases A-F. While SHAP values are unique to each model and absolute values cannot be directly compared between models, values within each model can be compared and relative ordering between models can be evaluated. *ACEi* angiotensin converting enzyme inhibitor, *CNS* central nervous system, *CPB* cardiopulmonary bypass, *DHCA* deep hypothermic circulatory arrest, *EDVP* end diastolic ventricular pressure, *ECMO* extracorporeal membrane oxygenation, *GI* gastrointestinal, *HCT* hematocrit, *ICU* intensive care unit, *PV* pulmonary vein, *PVR* pulmonary vascular resistance, *RCP* regional cerebral perfusion, *S1P* Stage 1 palliation, *S2P* Stage 2 palliation, *SHAP* SHapley Additive explanation, *TV* tricuspid valve, *UF* ultrafiltration.

**Figure 4:**
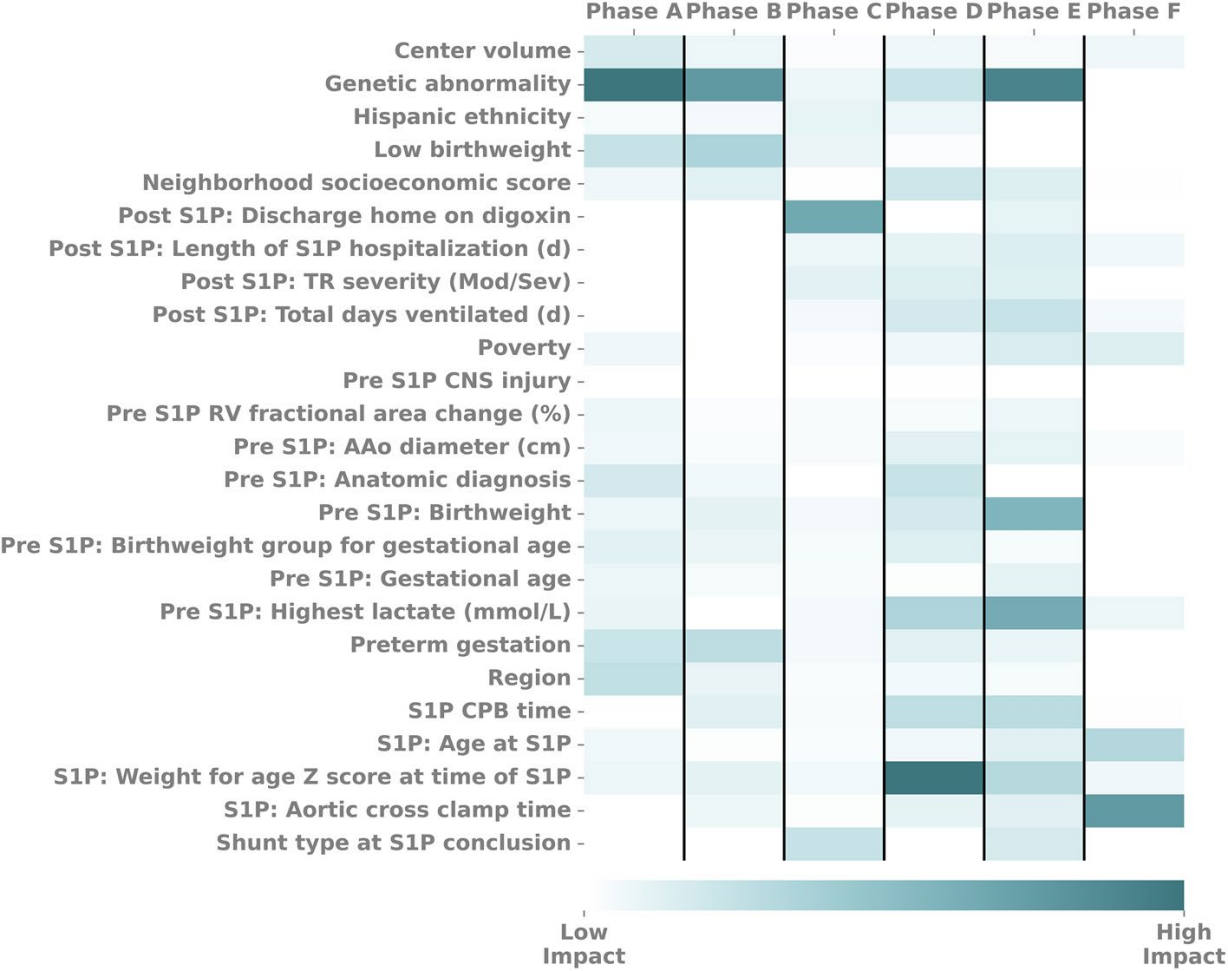
Heatmap showing the normalized, mean SHAP values for features commonly found throughout all 6 phases. Values are normalized within each model and then compared. Normalized values indicate changes in relative effect on the model in each stage. *AAo* ascending aorta, *CNS* central nervous system, *CPB* cardiopulmonary bypass, *RV* right ventricle, *S1P* Stage 1 palliation, *SHAP* SHapley Additive explanation, *TR* tricuspid regurgitation.

SHAP values associated with impactful features for models corresponding to Phase A through Phase F are summarized in Fig. 3. While intraoperative ECMO was the most important feature noted in the Phase B model, genetic and extracardiac anomalies, along with surgeon volume, low birthweight and preterm gestation remained significant contributors to model performance for both Phases A and B. Conversely, many Phase C features were driven by status at discharge (discharge home on antiarrhythmic, digoxin, or angiotensin converting enzyme therapy, oxygen saturation at discharge, supplemental oxygen at discharge, and shunt type at discharge). Phase D (interstage) model performance was driven by pre S1P (weight for age Z score at S1P, highest pre-S1P lactate), S1P (S1P cardiopulmonary bypass time) and post S1P features (interstage catheterization pulmonary vascular resistance and end diastolic ventricular pressure). Phase E (S2P) features again included the presence of genetic and extracardiac anomalies, along with post-S1P (CNS injury) and interstage (arrhythmia requiring therapy, and pulmonary vein saturation during interstage catheterization) features. Figure 4 summarizes mean SHAP values for features commonly found across all 6 phases and normalized within each model, indicating changes in relative feature effect on the model in each phase.

## Discussion

We report here a novel, individualized predictor of death or transplant through five years among patients with HLHS following S1P, incorporating both modifiable and nonmodifiable features known in the pre-S1P phase of care into an ML-driven predictive model. We also report a series of adaptive models which capture the evolution of individual risk by accounting for nonlinear effects of features associated with successive stages of care, with particularly robust model performance incorporating features through S1P hospitalization.

There are numerous studies reporting both modifiable and nonmodifiable risk factors associated with outcomes following S1P for HLHS. Previous efforts at predicting outcomes following S1P utilizing results from the PHN SVR trial to date have employed Cox proportional hazards or logistic regression models, largely focusing upon endpoints associated with the S1P hospitalization itself. Gupta et al. reported a model predicting mortality during S1P hospitalization with an area under the receiver operating characteristic curve (AUROC) of 0.77 and a poor composite outcome during S1P hospitalization (AUROC of 0.72), though these models incorporated intraoperative features thus rendering its utility for pre-S1P planning of limited value^7^. More recently, Jalali et al. reported the application of a deep learning model to pre-S1P features alone in predicting death or transplant beyond the S1P hospitalization to 1 year following S1P with an accuracy of 89 ± 4%^6^. Leveraging data from 3267 infants discharged home following S1P in the National Pediatric Cardiology Quality Improvement Collaborative, Sunthankar and colleagues reported the application of ML modeling to the prediction of interstage mortality specifically, with modest model performance (AUC 0.642 [95% CI 0.626–0.658])^8^. Furthermore, none of these studies evaluated likelihood of TFS at any time point beyond the SVR trial endpoint of 14 months. Given the longer-term burdens associated with a staged palliative approach to HLHS, the PHN SVR II study explored longer-term post S1P outcomes to 6 years of age with respect to shunt type specifically, and while demonstrating a higher incidence of catheter-based interventions in the RV2PAS subgroup, there was no significant difference in TFS at 6 years of age^5^. A subsequent analysis using restricted mean survival times and accounting for shunt type received during S1P (accounting for crossover following randomization) demonstrated superior TFS through five years of follow up among those with an RV2PAS (p = 0.033)^9^. Beyond the original SVR trial endpoint, there were an additional 40 patients (10.6%) of those event-free at 1 year (n = 377) meeting an endpoint of either transplant or death by 6 years of age. Therefore, a model that could render an individualized prediction of death or transplant well into childhood, with features known even prior to the initial S1P is an attractive tool to counsel families and may provide insight into the longer-term implications of a staged palliative approach to a lifelong condition. Employing only features available in the pre-S1P phase (Phase A), we report here a model predicting six-year TFS with satisfactory clinical performance with a C index of 0.692 (95% CI 0.690–0.695).

Beyond Phase A however, the adaptive nature of the modeling reported here provides an enhanced and individualized approach to understanding and adjusting risk over the course of staged palliative interventions for hypoplastic left heart syndrome. With successive phases of care comes additional clinical information and an evolution in an individual patient’s risk for death or transplant. This ML-driven modeling approach allows for the mapping of risk factors concurrently, accounting for nonlinear effects and interactions between features on an individualized basis, while also allowing for the removal of risk factors that may not remain relevant in a subsequent phase. Indeed, the conclusion of Phase C, the phase at greatest risk for death or transplant during the study period, also consisted of a sample size relatively larger than subsequent phases, and results in highest performance of the model series (C index 0.822 [95% CI 0.820–0.825]), performance that is preserved through the five-year study period. In subsequent clinical phases, model Brier scores approach 0, indicating that while the incorporation of more clinical information moderately improves the correct ordering of information, (as measured by the concordance index), the error of the prediction is progressively improved. With subsequent phases (D through F) and a decline in study subjects available for incorporation in both the train and test datasets however, class imbalances were associated with an overall gradual degradation in model performance.

The adaptive nature of the models reported here allows for identification of relative feature importance within each model as summarized in Fig. 3. In Phase A, model features are largely non-modifiable (preterm gestation, region, genetic abnormality), though arguably modifiable features including surgeon volume and preoperative enteral feeding also demonstrated significant contribution to this pre S1P model. Features identified at the time of S1P hospitalization discharge (specific discharge medications, supplemental oxygen, and shunt type) were of importance in the Phase C model, which also was the highest-performing model. Medical insurance status was of greater importance within the interstage (Phase D) model, more so than interstage pulmonary vascular resistance. We also demonstrate that within subsequent models, features apparent in Phase A (genetic abnormality, extracardiac anomaly, highest lactate pre S1P and age at S1P) remain of substantive importance to model performance across several phases.

To our knowledge this is the first adaptive model designed to adjust inputs in response to features associated with subsequent phases in a staged palliation. Despite its novelty, there are limitations inherent to this secondary analysis. First given the relatively small sample size, class imbalances led to degradation in model performance as patients progressed beyond their S1P hospitalization. Subsequent longitudinal studies of even larger populations with comparable feature numbers may provide a more robust and reproducible series of adaptive models. Second, while our series of models incorporated more features than traditional survival analyses, this series was limited by data only obtained as a part of the original SVR and SVRII studies; leveraging the processing power of ML-driven models will enable the analysis of far larger and more granular datasets and is an area for future exploration. Third, data missingness may have introduced bias into the models, particularly among models derived from increasingly diminutive populations.

Using ML-based survival analysis of publicly available longitudinal data from a randomized controlled clinical trial, we report the development of an individualized series of models predicting the probability of transplant-free survival through five years following S1P. We report the application of features to subsequent palliative stages of care for HLHS resulting in progressive improvements in model performance through the S1P hospitalization. Models derived from this process provide an individualized and adaptive means of predicting the probability of TFS and provides a roadmap for ML-driven clinical decision support in the management of complex congenital heart disease.

## Methods

### Data source

From 2005 through 2009, participants undergoing a S1P at one of 15 participating centers meeting the inclusion criteria of HLHS or related single, morphologic systemic right ventricular anatomy were considered for enrollment in the PHN SVR trial, with 549 patients meeting eligibility criteria, undergoing randomization, and ultimately undergoing either a modified Blalock-Taussig-Thomas shunt (mBTTs) or a right ventricle to pulmonary artery (RV2PA) conduit as the source of pulmonary blood flow as a component of their S1P. Trial design and results of the primary analysis have previously been reported^4,10^. The original protocol was approved by an independent Protocol Review Committee and by the institutional review boards of each participating clinical center, with written informed consent from parent(s) or legal guardians obtained at respective centers prior to SVR trial enrollment; this secondary analysis of publicly available, de-identified SVR study data was also prospectively acknowledged by the Johns Hopkins Medicine Institutional Review Board (IRB# 00318251). All analyses were performed in accordance with relevant guidelines and regulations.

The primary outcome measure of interest was TFS. Using data from the SVR trial, model design relied upon the classification of input features with respect to sequential time points along an infant’s course of care. Model features, including those based upon previously-established clinical relevance and subject matter expert opinion, were included in an approach comparable to previous studies analyzing data collected during the course of the original SVR trial^6,11–13^. Features available in the time period immediately prior to S1P (Phase A) from the SVR trial were employed in the training of the original model (Supplemental Table S1). Subsequent phases of care were assigned with respect to clinically relevant transitions in care. The Phase B model included patients undergoing S1P (operative conditions) in addition to features from Phase A. Phase C modeling included patients surviving Phase B, and included features associated with inpatient postoperative convalescence following S1P along with those from Phases A and B. Phase D modeling included all patients surviving to S1P hospitalization discharge prior to S2P, and considered features reported following S1P hospitalization discharge until S2P in addition to features from Phases A through C. Those surviving S1P but not discharged home prior to S2P palliation represented a relatively small (n = 22) subset of patients exposed to substantively different environmental conditions while hospitalized during the interstage period; this subgroup was accordingly excluded from consideration in the Phase D model. Phase E modeling included all patients undergoing S2P, and considered operative features specific to S2P in addition to consideration of features from Phases A through D. Finally, Phase F modeling included all patients who successfully completed S2P and included features associated with inpatient postoperative convalescence following S2P, in addition to features from Phases A through E. Features included within each model are described in Supplemental Table S1.

### ML analysis

Survival analysis entails the prediction of the probability of an event occurring over time. ML models demonstrate higher performance than traditional methods for survival analysis and represent a promising approach to incorporating more features into a survival model^14^. Traditional survival analysis uses the proportional hazards model in which the partial likelihood of an event is determined as a product of the likelihoods. Leveraging ML, the partial likelihood is replaced with the output of an ML model and the proportional hazards calculated with that output. This enables the capture of more complex, non-linear relationships compared to traditional methods along with a larger set of features. Models were built using the scikit-survival package^15^.

### Data preparation

Preparation of data for model training consisted of several steps. Data was classified as either numerical or categorical. For categorical data, values were either classified as ordinal or label. The former was encoded using an ordinal encoder, while the latter were encoded using a label encoder. In a few cases, values were binarized when ~ 50% or more of the values were in a single category and remaining values were distributed over several other categories. Data identified as numerical was encoded using either min–max scaling (normalization) or standard scaling (standardization). To preserve the original distributions, variables that appeared normal were standardized, while variables that appeared evenly distributed over a range were normalized.

Following data preparation, missing values were imputed using the following iterative approach:

1. Missing values are filled by random sampling from the observed data.
2. The first variable (x_1_) is regressed on all other variables (x_2_,…,x_k_) with observed x_1_.
3. Missing values for x_1_ are replaced using the distribution for x_1_ generated in step 2.
4. Iterate over each variable until complete.

Missing forest has demonstrated better results compared to traditional multiple imputation with chained equations for medical datasets^16^. As a result, a singly imputed dataset using random forest was used.

Feature selection was accomplished by a combination of univariate feature selection using mutual information score of each feature as compared to the survival time of the patient, followed by sequential feature selection. A range of features were selected using univariate selection and then models were built in which those selected features were sequentially eliminated, and the model score recorded. For each iteration, the feature resulting in the smallest score change was removed. This was repeated until the pre-determined number of features had been achieved. Short optimization runs were then performed for each feature set, and the highest performing run was selected for further optimization.

### Model training

Models consisted of a set of parameters (learned from model training) and hyperparameters (set by the user). Hyperparameter tuning to identify optimal hyperparameters was performed using Bayesian optimization. To prevent overfitting of the model (high performance on the test set, but low performance on the test set), a fivefold cross validation was used.

### Model evaluation

Metrics for evaluation of a survival model include the concordance index, Brier score and time-dependent area under the curve (td-AUC). The concordance index is a measure of the correct ordering of the predicted time series compared to the actual time series, with values ≥ 0.6 commonly reported for medical datasets^17–19^. The Brier score is analogous to the mean squared error of the predicted survival time, with values ≤ 0.20 typically reported for medical datasets^20,21^. The td-AUC is the traditional area under the curve at a given time point. As predictions change with time, the td-AUC is calculated over a range of time points and measures the change in model performance over a given period of time (i.e. how far out can the model predict while still maintaining sufficient performance), with a td-AUC > 0.70 considered sufficiently performant for clinical applications^22^.

### Explainable artificial intelligence (XAI)

Model explanation refers to a quantitative relationship between the value of an input feature and the predicted output. SHapley Additive exPlanations (SHAP) values represent a game theoretic approach to determine the impact of model features on a prediction^23^. SHAP values quantify the impact of a feature, by determining the value of a prediction with that feature compared to the value of the prediction if the feature took some baseline value. Individual feature contributions can be calculated as the difference between a model output with a feature compared to the baseline value when that feature is replaced with the expected value.

### Supplementary Information

Supplementary Table 1.

## Data Availability

The datasets analyzed during the current study are available in the Pediatric Heart Network Repository, https://www.pediatricheartnetwork.org/public-use-data-sets/.

## Abbreviations

AAo: Ascending aorta
ACEi: Angiotensin converting enzyme inhibitor
AoV: Aortic valve
ASD: Atrial septal defect
AV: Atrioventricular
AVV: Atrioventricular valve
CNS: Central nervous system
CPB: Cardiopulmonary bypass
CPR: Cardiopulmonary resuscitation
CVA: Cerebrovascular accident
DHCA: Deep hypothermic circulatory arrest
ECMO: Extracorporeal membrane oxygenation
GI: Gastrointestinal
HCT: Hematocrit
HLHS: Hypoplastic left heart syndrome
ICH: Intracranial hemorrhage
ICU: Intensive care unit
LOS: Length of stay
LPA: Left pulmonary artery
mBTTS: Modified Blalock-Taussig-Thomas shunt
ML: Machine learning
MV: Mitral valve
NeoAI: Neoaortic valve insufficiency
PA: Pulmonary artery
PAPVR: Partial anomalous pulmonary venous return
PHN: Pediatric Heart Network
PM: Pacemaker
PV: Pulmonary vein
PVR: Pulmonary vascular resistance
RCP: Regional cerebral perfusion
RPA: Right pulmonary artery
RSCA: Right subclavian artery
RV: Right ventricle
RV2PA: Right ventricle to pulmonary artery
S1P: Stage 1 palliation
S2P: Stage 2 palliation
SHAP: SHapley Additive exPlanations
SVC: Superior vena cava
SVR: Single Ventricle Reconstruction
TAPVR: Total anomalous pulmonary venous return
td-AUC: Time-dependent area under the curve
TFS: Transplant-free survival
TR: Tricuspid valve regurgitation
TV: Tricuspid valve
UF: Ultrafiltration
