# Supplementary material for: Using machine learning to predict five-year transplant-free survival among infants with hypoplastic left heart syndrome
Source: Sci Rep. 2024 Feb 24;14:4512. doi: 10.1038/s41598-024-55285-1 (PMC10894293; doi:10.1038/s41598-024-55285-1)
Supplement: Supplementary file 1 — Supplementary Table 1. [file 41598_2024_55285_MOESM1_ESM.docx]

**Using Machine Learning To Predict Five-Year Transplant-Free Survival Among Infants with Hypoplastic Left Heart Syndrome**

**Authors**

Andrew H. Smith MD MSCI MMHC^1^, Geoffrey M. Gray PhD^2,5^, Awais Ashfaq MD^3^, Alfred Asante-Korang MD^4^, Mohamed A. Rehman MD^2,5^, Luis M. Ahumada PhD^2,5^

**Affiliations**

1. Division of Cardiac Critical Care Medicine, The Heart Institute, Johns Hopkins All Children’s Hospital; St. Petersburg FL.
2. Center for Pediatric Data Science and Analytic Methodology, Johns Hopkins All Children's Hospital, St. Petersburg, FL.
3. Cardiovascular Surgery, Heart Institute, Johns Hopkins All Children's Hospital, St. Petersburg, FL.
4. Heart Transplantation, Cardiomyopathy and Heart Failure, Heart Institute, Johns Hopkins All Children's Hospital, St. Petersburg, FL.
5. Department of Anesthesia and Pain Medicine, Johns Hopkins All Children's Hospital, St. Petersburg, FL.

Corresponding author:

Andrew Harold Smith, MD MSCI MMHC
Division of Cardiac Critical Care Medicine
The Heart Institute

Johns Hopkins All Children’s Hospital

501 6^th^ Avenue South

St. Petersburg, FL 33701

727.767.8576 (p)

[asmit356@jhmi.edu](mailto:asmit356@jhmi.edu)

**Supplemental Table 1.** Features incorporated into the model design with respect to phase of care.

|  | **Model Phase** | | | | | |
| --- | --- | --- | --- | --- | --- | --- |
| **Feature** | **A** | **B** | **C** | **D** | **E** | **F** |
| AAo diameter (Pre S1P) | X | X | X | X | X | X |
| Age at S1P | X | X | X | X | X | X |
| APGAR at 1 minute | X | X | X | X | X | X |
| Birthweight | X | X | X | X | X | X |
| Birthweight percentile for gestational age | X | X | X | X | X | X |
| Center volume | X | X | X | X | X | X |
| Extracardiac anomaly identified | X | X | X | X | X | X |
| Genetic abnormality | X | X | X | X | X | X |
| Gestational age | X | X | X | X | X | X |
| Medical insurance type | X | X | X | X | X | X |
| Neighborhood Socioeconomic Score | X | X | X | X | X | X |
| Number of complications (Pre S1P) | X | X | X | X | X | X |
| Preterm gestation | X | X | X | X | X | X |
| Region | X | X | X | X | X | X |
| RV fractional area change (Pre S1P) | X | X | X | X | X | X |
| Surgeon volume (S1P) | X | X | X | X | X | X |
| Weight for age Z score at time of S1P | X | X | X | X | X | X |
| Low birthweight | X | X | X | X | X |  |
| Intubation indication apnea or transport (Pre S1P) | X | X | X | X |  | X |
| Hispanic ethnicity | X | X | X | X |  |  |
| Aortic arch interruption | X | X | X |  |  | X |
| Left ventricle present | X | X |  | X | X |  |
| MV regurgitation present (Pre S1P) | X | X |  | X | X |  |
| MV regurgitation severity (Pre S1P) | X | X |  | X | X |  |
| Anatomic diagnosis | X | X |  | X |  | X |
| Anomalous pulmonary venous return | X | X |  | X |  |  |
| Pre S1P noncardiac surgery | X | X |  | X |  |  |
| Age at diagnosis | X | X |  |  |  |  |
| APGAR at 5 minutes | X |  | X | X | X | X |
| Highest lactate (Pre S1P) | X |  | X | X | X | X |
| Poverty score | X |  | X | X | X | X |
| Aortic atresia | X |  | X | X | X |  |
| Enteral feeds prior to S1P | X |  | X | X |  | X |
| Inhaled N_2_ (Pre S1P) | X |  | X | X |  | X |
| Intubation (Pre S1P) | X |  | X | X |  |  |
| AoV regurgitation present (Pre S1P) | X |  | X |  |  | X |
| Shock (Pre S1P) | X |  | X |  |  | X |
| TV regurgitation severity (Pre S1P) | X |  | X |  |  |  |
| TV regurgitation present (Pre S1P) | X |  |  | X | X |  |
| AoV regurgitation severity (Pre S1P) | X |  |  |  |  | X |
| Intubation indication shock or respiratory failure (Pre S1P) | X |  |  |  |  | X |
| Systemic venous anomaly(s) | X |  |  |  |  | X |
| **Feature** | **A** | **B** | **C** | **D** | **E** | **F** |
| Fetal intervention | X |  |  |  |  |  |
| Prenatal diagnosis | X |  |  |  |  |  |
| Shunt type intended for S1P (Pre S1P) | X |  |  |  |  |  |
| Aortic cross clamp time (S1P) |  | X | X | X | X | X |
| CNS injury (Pre S1P) |  | X | X | X | X | X |
| CPB time (S1P) |  | X | X | X | X | X |
| Deep hypothermic circulatory arrest time (S1P) |  | X | X | X | X | X |
| Lowest hematocrit during surgery (S1P) |  | X | X | X | X | X |
| Lowest temperature during surgery (S1P) |  | X | X | X | X | X |
| Pulmonary venous obstruction (Pre S1P) |  | X | X | X | X |  |
| Associated anatomic diagnoses (Pre S1P) |  | X | X | X |  | X |
| ECMO in OR (S1P) |  | X | X | X |  | X |
| Intraoperative anatomy: TAPVR (S1P) |  | X | X | X |  | X |
| Procedures concurrent with S1P: Branch PA plasty |  | X | X | X |  | X |
| Procedures concurrent with S1P: PM implant |  | X | X | X |  | X |
| Inhaled CO_2_ (Pre S1P) |  | X | X | X |  |  |
| Intraoperative anatomy: aberrant RSCA (S1P) |  | X | X | X |  |  |
| Procedures concurrent with S1P: Repair TAPVR |  | X | X | X |  |  |
| Alpha blockade utilized (S1P) |  | X | X |  | X | X |
| Intraoperative anatomy: PAPVR (S1P) |  | X | X |  |  |  |
| Cardiac surgery (Pre S1P) |  | X |  | X | X | X |
| Continuous ultrafiltration used after CPB (S1P) |  | X |  | X | X | X |
| Deep hypothermic circulatory arrest used (S1P) |  | X |  | X | X |  |
| Pre S1P catheterization |  | X |  | X | X |  |
| Continuous ultrafiltration used during CPB (S1P) |  | X |  |  |  |  |
| Age at S1P hospitalization discharge (Post S1P) |  |  | X | X | X | X |
| AVV dysfunction (Post S1P) |  |  | X | X | X | X |
| Bowel surgery (Post S1P) |  |  | X | X | X | X |
| Chronic respirtatory failure (Post S1P) |  |  | X | X | X | X |
| Chylothorax (Post S1P) |  |  | X | X | X | X |
| Diaphragm plication (Post S1P) |  |  | X | X | X | X |
| Discharge home on digoxin (Post S1P) |  |  | X | X | X | X |
| Gastrostomy placed (Post S1P) |  |  | X | X | X | X |
| Gender |  |  | X | X | X | X |
| GI complication (Post S1P) |  |  | X | X | X | X |
| ICU days (Post S2P) |  |  | X | X | X | X |
| Infectious complication (Post S1P) |  |  | X | X | X | X |
| Length of S1P hospitalization |  |  | X | X | X | X |
| LPA hypoplasia (Post S1P) |  |  | X | X | X | X |
| NeoAI present (Post S1P) |  |  | X | X | X | X |
| Number of complications (Post S1P) |  |  | X | X | X | X |
| Number of interventional catheterizations (Post S1P) |  |  | X | X | X | X |
| Number of medications at discharge (Post S1P) |  |  | X | X | X | X |
| Number of serious adverse events prior to S1P discharge |  |  | X | X | X | X |
| Number of surgical procedures (Post S1P) |  |  | X | X | X | X |
| O2 saturation at discharge (Post S1P) |  |  | X | X | X | X |
| PA reconstruction (Post S1P) |  |  | X | X | X | X |
| Pacemaker implant (Post S1P) |  |  | X | X | X | X |
| **Feature** | **A** | **B** | **C** | **D** | **E** | **F** |
| Phrenic nerve injury (Post S1P) |  |  | X | X | X | X |
| Regional cerebral perfusion time (S1P) |  |  | X | X | X | X |
| Renal failure (Post S1P) |  |  | X | X | X | X |
| RPA hypoplasia (Post S1P) |  |  | X | X | X | X |
| RV fractional area change (Post S1P) |  |  | X | X | X | X |
| Shunt type at S1P conclusion |  |  | X | X | X | X |
| Shunt revision or crossover (Post S1P) |  |  | X | X | X | X |
| Total days ventilated (Post S1P) |  |  | X | X | X | X |
| TR present (Post S1P) |  |  | X | X | X | X |
| TR severity (Post S1P) |  |  | X | X | X | X |
| Tracheostomy (Post S1P) |  |  | X | X | X | X |
| Procedures concurrent with S1P: AVV plasty |  |  | X | X | X |  |
| Discharge home on antiarrhyrthmic (Post S1P) |  |  | X | X |  | X |
| ECMO (Post S1P) |  |  | X | X |  | X |
| Procedures concurrent with S1P: Repair PAPVR |  |  | X | X |  | X |
| Procedures concurrent with S1P: Any other |  |  | X | X |  |  |
| CNS injury (Post S1P) |  |  | X |  | X | X |
| Vocal cord dysfunction (Post S1P) |  |  | X |  | X | X |
| Discharge home on ACEi (Post S1P) |  |  | X |  | X |  |
| Race |  |  | X |  | X |  |
| Aprotinin used (S1P) |  |  | X |  |  | X |
| Interventional catheterization following S1P |  |  | X |  |  | X |
| Open sternum (Post S1P) |  |  | X |  |  | X |
| Supplemental oxygen at discharge (Post S1P) |  |  | X |  |  | X |
| Venous thromboembolism (Post S1P) |  |  | X |  |  | X |
| Any arrhythmia requiring therapy (Post S1P) |  |  | X |  |  |  |
| AV block (Post S1P) |  |  | X |  |  |  |
| CPR (Post S1P) |  |  | X |  |  |  |
| RV dysfunction (Post S1P) |  |  | X |  |  |  |
| Age at start of Phase D |  |  |  | X | X | X |
| Aortic saturation (interstage catheterization) |  |  |  | X | X | X |
| Arrhythmia requiring therapy (interstage) |  |  |  | X | X | X |
| AV block (interstage) |  |  |  | X | X | X |
| AVV insultrafiltrationficiency requiring therapy (interstage) |  |  |  | X | X | X |
| Chronic respirtatory failure (interstage) |  |  |  | X | X | X |
| Chylothorax (interstage) |  |  |  | X | X | X |
| Diaphragm paralysis (interstage) |  |  |  | X | X | X |
| End diastolic ventricular pressure (interstage catheterization) |  |  |  | X | X | X |
| GI complication (interstage) |  |  |  | X | X | X |
| Infectious complication (interstage) |  |  |  | X | X | X |
| Mean arterial pressure (interstage catheterization) |  |  |  | X | X | X |
| Mean systemic venous atrial pressure (interstage catheterization) |  |  |  | X | X | X |
| Neuro complication (ICH, Seizure, CVA) (interstage) |  |  |  | X | X | X |
| PV saturation (interstage catheterization) |  |  |  | X | X | X |
| PVR (interstage catheterization) |  |  |  | X | X | X |
| Qp:Qs (interstage catheterization) |  |  |  | X | X | X |
| **Feature** | **A** | **B** | **C** | **D** | **E** | **F** |
| Renal failure (interstage) |  |  |  | X | X | X |
| RV dysfunction (interstage) |  |  |  | X | X | X |
| SVC saturation (interstage catheterization) |  |  |  | X | X | X |
| Systemic ventricular pressure (interstage catheterization) |  |  |  | X | X | X |
| Vocal cord dysfunction (interstage) |  |  |  | X | X | X |
| Venous thromboembolism (interstage) |  |  |  | X | X | X |
| AM start (S1P) |  |  |  | X | X |  |
| Arrhythmia requiring therapy (Pre S1P) |  |  |  | X |  |  |
| ASD restriction (Pre S1P) |  |  |  | X |  |  |
| Regional cerebral perfusion (S1P) |  |  |  | X |  |  |
| Steroids administered (S1P) |  |  |  | X |  |  |
| Abnormal pulmonary venous drainage (Pre S2P) |  |  |  |  | X | X |
| Abnormal systemic venous drainage (Pre S2P) |  |  |  |  | X | X |
| Age at start of Phase E |  |  |  |  | X | X |
| Alpha blockade utilized (S2P) |  |  |  |  | X | X |
| AoV regurgitation (Pre S2P) |  |  |  |  | X | X |
| Aprotinin used (S2P) |  |  |  |  | X | X |
| Arch obstruction (Pre S2P) |  |  |  |  | X | X |
| Arch repair (S2P) |  |  |  |  | X | X |
| ASD restriction requiring intervention (Pre S2P) |  |  |  |  | X | X |
| Diaphragm plication (S2P) |  |  |  |  | X | X |
| Repair of RV2PA shunt complication (S2P) |  |  |  |  | X | X |
| AVV insufficiency (Pre S2P) |  |  |  |  | X | X |
| AVV repair or replacement (S2P) |  |  |  |  | X | X |
| Bowel surgery (interstage) |  |  |  |  | X | X |
| Branch PA stensosis (Pre S2P) |  |  |  |  | X | X |
| CPB time (S2P) |  |  |  |  | X | X |
| CPB used (S2P) |  |  |  |  | X | X |
| Deep hypothermic circulatory arrest time (S2P) |  |  |  |  | X | X |
| Deep hypothermic circulatory arrest used (S2P) |  |  |  |  | X | X |
| Diaphragm plication (interstage) |  |  |  |  | X | X |
| ECMO (interstage) |  |  |  |  | X | X |
| ECMO in OR (S2P) |  |  |  |  | X | X |
| Gastrostomy placed (interstage) |  |  |  |  | X | X |
| Lowest hematocrit (S2P) |  |  |  |  | X | X |
| Lowest temperature (S2P) |  |  |  |  | X | X |
| NeoAI (Pre S2P) |  |  |  |  | X | X |
| Number of other surgical procedures (interstage) |  |  |  |  | X | X |
| Number of Pre S2P interventional catheterizations |  |  |  |  | X | X |
| PA plasty or dilation or stent placement (S2P) |  |  |  |  | X | X |
| Pacemaker (S2P) |  |  |  |  | X | X |
| Pacemaker implant (interstage) |  |  |  |  | X | X |
| Pulmonary vein obstruction (Pre S2P) |  |  |  |  | X | X |
| RCP time (S2P) |  |  |  |  | X | X |
| RCP used (S2P) |  |  |  |  | X | X |
| Reason for S2P timing: Moderate or greater AVV insufficiency |  |  |  |  | X | X |
| Reason for S2P timing: Neoaortic obstruction |  |  |  |  | X | X |
| **Feature** | **A** | **B** | **C** | **D** | **E** | **F** |
| Repair or revision of anomalous pulmonary veins (S2P) |  |  |  |  | X | X |
| S2P reason for timing: Elective |  |  |  |  | X | X |
| S2P reason for timing: Failure to thrive |  |  |  |  | X | X |
| S2P reason for timing: PA stenosis |  |  |  |  | X | X |
| S2P reason for timing: Progressive hypoxemia |  |  |  |  | X | X |
| S2P reason for timing: Shunt occlusion |  |  |  |  | X | X |
| S2P reason for timing: Shunt stenosis |  |  |  |  | X | X |
| S2P reason for timing: Ventricular dysfunction |  |  |  |  | X | X |
| S2P Surgical Type |  |  |  |  | X | X |
| Steroids (S2P) |  |  |  |  | X | X |
| Supplemental oxygen at time of S2P admission |  |  |  |  | X | X |
| Ultrafiltration used during CPB (S2P) |  |  |  |  | X | X |
| Ultrafiltration used during post-CPB (S2P) |  |  |  |  | X | X |
| Arrhythmia requiring therapy (Post S2P) |  |  |  |  |  | X |
| AV block (Post S2P) |  |  |  |  |  | X |
| AVV dysfunction requiring therapy (Post S2P) |  |  |  |  |  | X |
| Bowel surgery (Post S2P) |  |  |  |  |  | X |
| Chronic respiratory failure (Post S2P) |  |  |  |  |  | X |
| Chylothorax (Post S2P) |  |  |  |  |  | X |
| CPR (Post S2P) |  |  |  |  |  | X |
| Duration MV (Post S2P) |  |  |  |  |  | X |
| ECMO (Post S2P) |  |  |  |  |  | X |
| Extubated in OR (S2P) |  |  |  |  |  | X |
| Gastrostomy tube (Post S2P) |  |  |  |  |  | X |
| GI complication (Post S2P) |  |  |  |  |  | X |
| Home on ACEi (Post S2P) |  |  |  |  |  | X |
| Home on antiarrhythmic (Post S2P) |  |  |  |  |  | X |
| Home on digoxin (Post S2P) |  |  |  |  |  | X |
| Hospital LOS (Post S2P) |  |  |  |  |  | X |
| ICU days (Post S2P) |  |  |  |  |  | X |
| Infectious complication (Post S2P) |  |  |  |  |  | X |
| Infectious complication (Pre S1P) |  |  |  |  |  | X |
| Interventional cath (Post S2P) |  |  |  |  |  | X |
| Neuro complication (Post S2P) |  |  |  |  |  | X |
| Number of discharge medications (Post S2P) |  |  |  |  |  | X |
| Number of interventional catheterization procedures (Post S2P) |  |  |  |  |  | X |
| Number of other surgical procedures (Post S2P) |  |  |  |  |  | X |
| Number of significant postoperative complications (Post S2P) |  |  |  |  |  | X |
| Operative revision with CPB (S2P) |  |  |  |  |  | X |
| Oxygen saturation at discharge (Post S2P) |  |  |  |  |  | X |
| Pacemaker implant (Post S2P) |  |  |  |  |  | X |
| Phrenic Nerve Injury (Post S2P) |  |  |  |  |  | X |
| Diaphragm plication (Post S2P) |  |  |  |  |  | X |
| Renal failure (Post S2P) |  |  |  |  |  | X |
| RV dysfunction (Post S2P) |  |  |  |  |  | X |
| Home on supplemental oxygen (Post S2P) |  |  |  |  |  | X |
| **Feature** | **A** | **B** | **C** | **D** | **E** | **F** |
| Vocal cord dysfunction (Post S2P) |  |  |  |  |  | X |
| Venous thromboembolism (Post S2P) |  |  |  |  |  | X |

AAo: ascending aorta; ACEi: angiotensin converting enzyme inhibitor; AoV: aortic valve; AoV: aortic valve; ASD: atrial septal defect; AV: atrioventricular; AVV: atrioventricular valve; CNS: central nervous system; CPB: cardiopulmonary bypass; CPR: cardiopulmonary resuscitation; CVA: cerebrovascular accident; ECMO: extracorporeal membrane oxygenation; GI: gastrointestinal; ICH: intracranial hemorrhage; ICU: intensive care unit; LOS: length of stay; LPA: left pulmonary artery; MV: mitral valve;

NeoAI: neoaortic valve insufficiency; PA: pulmonary artery; PAPVR: partial anomalous pulmonary venous return; PM: pacemaker; PV: pulmonary vein; PVR: pulmonary vascular resistance; RCP: regional cerebral perfusion; RPA: right pulmonary artery;

RSCA: Right subclavian artery; RV: right ventricle; RV2PA: right ventricle to pulmonary artery; S1P: Stage 1 palliation; S2P: Stage 2 palliation; SVC: superior vena cava; TAPVR: total anomalous pulmonary venous return; TR: Tricuspid valve regurgitation; TV: tricsupid valve
